# Anesthesia in patients with Brugada syndrome: two case reports

**DOI:** 10.1186/s13256-023-03934-w

**Published:** 2023-06-16

**Authors:** Che-Hao Hsu, Shin-Hong Lin, Li-Yen Chang

**Affiliations:** 1 Department of Anesthesiology, Tungs’ Taichung MetroHarbor Hospital, Taichung, 43503 Taiwan; 2 Department of Anesthesiology, Taichung Armed Forces General Hospital, Taichung, 41169 Taiwan

**Keywords:** Ventricular arrhythmia, Brugada syndrome

## Abstract

**Background:**

Brugada syndrome is a rare disease. It causes sudden cardiac arrest, which is a serious life-threatening event. Sudden cardiac death mostly results from coronary artery disease. However, patients with Brugada syndrome show normal cardiac anatomy and no evidence of ischemia or electrolyte imbalance. Anesthesia in patients with Brugada syndrome is challenging due to its unpredictable nature, and is worth our attention.

**Case presentation:**

We report two cases of Brugada syndrome during anesthesia. In case one, a 31-year-old Filipino laborer was scheduled for laparoscopic appendectomy. The patient denied any preexisting cardiac disease. The preoperative vital signs were stable, with mild fever of 37.9 °C. The operation was smooth. During the emergence period, the patient suffered from sudden onset of ventricular tachycardia. After resuscitation, the cardiac rhythm returned to normal. Later, he was confirmed to have a genetic trait of Brugada syndrome. In case two, a young Taiwanese patient with pre-diagnosed Brugada syndrome underwent an operation. The perioperative precautions were taken to prevent the occurrence of ventricular arrhythmia. The surgery was uneventful.

**Conclusions:**

Brugada syndrome, although rare, has the highest incidence in South East Asian healthy young males. It brings attention to possible fatal cardiac arrhythmia in this population. Careful preoperative evaluation and perioperative management can help reduce the harmful outcome of the disease and prevent any untoward events.

## Background

Brugada syndrome (BrS) is a rare disease. It causes sudden cardiac arrest (SCA), which is a serious and life-threatening event. SCA mostly results from coronary artery disease. However, patients with Brugada syndrome show normal cardiac anatomy and no evidence of ischemia or electrolyte imbalance. Brugada syndrome accounts for 4–20% of sudden cardiac death [1, 2]. These patients do not feel discomfort before the attack, but suffer syncope or sudden death [1]. Anesthesia in patients with Brugada syndrome is challenging due to its unpredictable nature. Here we report two cases of Brugada syndrome; one young patient without any preexisting cardiac disease suffered from sudden onset of ventricular tachycardia during anesthesia, and the other patient with prediagnosed Brugada syndrome underwent an uneventful surgery.

## Case 1

A 31-year-old Filipino laborer was scheduled for operation of appendectomy. He received laparoscopic appendectomy. No significant medical history was ever traced. The preoperative vital signs were stable, with mild fever of 37.9 °C.

General anesthesia was induced with fentanyl 100 μg, propofol 100 mg, and rocuronium 35 mg. Maintenance of anesthesia was achieved with sevoflurane 2.0% through 7.5Fr endotracheal tube. Fentanyl 50 μg and rocuronium 10 mg were also added intravenously every 30 minutes as maintenance. The operation was smooth, with minimal blood loss and stable vital signs. Total operation time was about 2 hours.

**Fig. 1:**
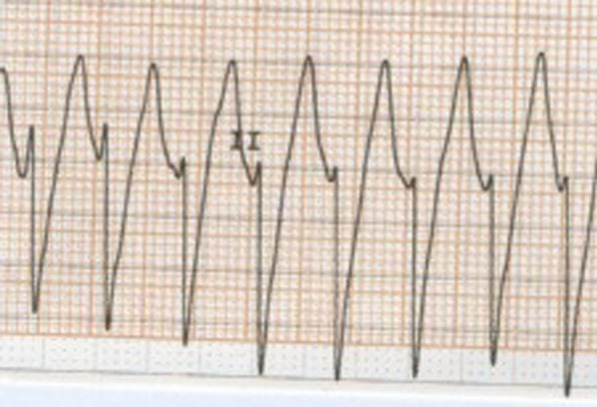
Intra-operation electrocardiogram lead II showing ventricular tachycardia

During the emergence period, after administration of the reverse agent (atropine 1 mg and neostigmine 2.5 mg), the patient was breathing spontaneously. Suddenly, the electrocardiogram (ECG) monitor showed ventricular tachycardia (Fig. 1). Blood pressure and pulse were undetected. Cardiopulmonary resuscitation (CPR) and defibrillation were initiated immediately. After given two doses of 1 mg intravenous epinephrine separated by a cycle of cardiac massage and one electric defibrillation, the ECG returned to normal sinus rhythm. His vital signs returned to the baseline level. Arterial blood gas analysis showed no severe metabolic acidosis.

**Fig. 2:**
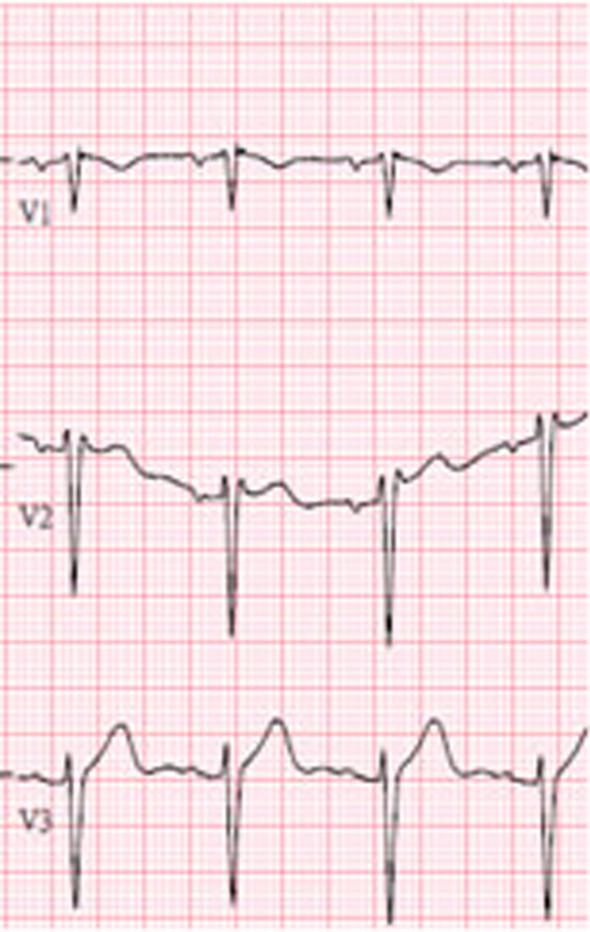
V2 lead showing saddleback-shaped pattern, suspect of Brugada syndrome

After this serious cardiac event, he was transferred to intensive care unit for observation. Cardiologist was also consulted. Further surveys including 12 lead ECG, ck-MB, troponin-T, cardiac echography, and C-reactive protein were carried out. None of them showed significant abnormalities except the 12 lead ECG done at post-recovery room, which showed coved-type ST elevation in lead V2 and V3. Saddleback pattern in lead V2 was also noted in ECG follow-up later that day (Fig. 2). Brugada syndrome was highly suspected.

After tracing his family history, there was no incidence of sudden death in his close relatives. He was discharged 4 days later and was referred to medical center for further study. Later he was confirmed to have genetic trait of Brugada syndrome.

## Case 2

A 20-year-old native Taiwanese patient was admitted due to compression injury to right hand by a heavy object at work. Surgical intervention was arranged.

According to the patient and his family, he suffered from Brugada syndrome. His father died of sudden cardiac arrest at the age of 36. After that tragic event, he was brought to National Taiwan University Hospital for Brugada syndrome genetic testing, which revealed he was positive for SCN5A gene mutation. He had not suffered any symptoms of Brugada syndrome before.

**Fig. 3:**
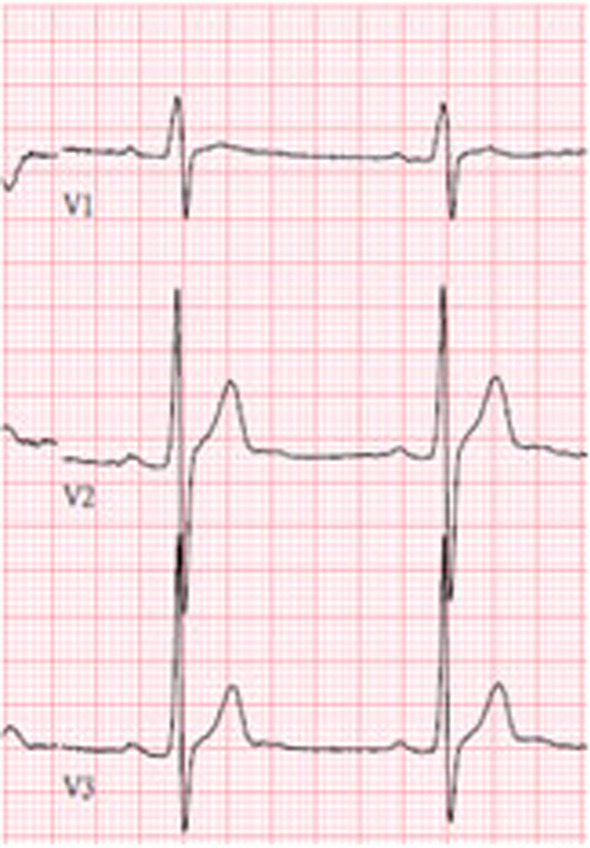
Typical Brugada electrocardiogram wave form not seen in case 2

Preoperation 12 lead ECG exam revealed sinus bradycardia. Typical Brugada ECG wave form was not seen (Fig. 3).

The patient’s anesthesia was induced with fentanyl 100 μg, thiamylal sodium 600 mg, and rocuronium 70 mg; lidocaine was avoided due to its QT prolonged effect. Arterial line was established for intraoperative blood pressure and electrolyte monitoring. External defibrillator was also prepared. The operation was smooth and lasted 1 hour, and vital signs were stable. After the operation, he was sent to our surgical intensive care unit (SICU) for observation, and later discharged.

## Discussion

### Epidemiology

Brugada syndrome varies significantly between gender, regions, and races. Although the suspected related gene is distributed equally between the sexes, the phenotype tends to be exhibited more severely in males, which makes the incidence in the male population (about 80%) much higher than in the female population [3, 4]. Worldwide pooled prevalence is 0.5 per 1000 [5]. The highest prevalence was reported in South East Asia, 1.8 per 1000, and the lowest in North Africa, 0 per 1000 [3, 4]. Generally, prevalence in Asians is 9 times higher than in Caucasians and 36 times higher than in Hispanics. Types II/III BrS are much more common than type I BrS, with worldwide prevalence of 6.1 per 1000 and 35.5 per 1000 in South East Asia [6, 7].

### Symptoms and clinical presentation

Brugada syndrome is a genetic disorder in which the cardiac electrical activity is abnormal. Most patients with BrS do not have any symptoms; however, it increases the chances of ventricular arrhythmias and sudden cardiac death. Those affected may have fainting episodes due to brief abnormal heart rhythms. If a dangerous heart rhythm does not stop, a fatal cardiac arrest may occur. The most significant clinical manifestations of BrS are ventricular arrhythmias, although these patients may also be at risk of atrial arrhythmias, most notably atrial fibrillation (AF). The incidence of AF is higher in patients with BrS, and the presence of AF has been associated with increased disease severity and a higher risk of ventricular fibrillation (VF). Moreover, fainting episodes can manifest even with a normal heart rhythm due to a sudden drop in blood pressure, and are usually mistaken for vasovagal syncope [8].

Brugada syndrome is also known as sudden unexplained nocturnal death syndrome, with arrhythmic events more common at night and during sleep. Syncope or sudden death may be the only symptom during the attack. Brugada syndrome can happen in all ages with the highest incidence between 40 and 45 years old; however, the first occurrence of symptoms may occur in early childhood or old age [9, 10].

### Diagnosis and types

**Fig. 4:**
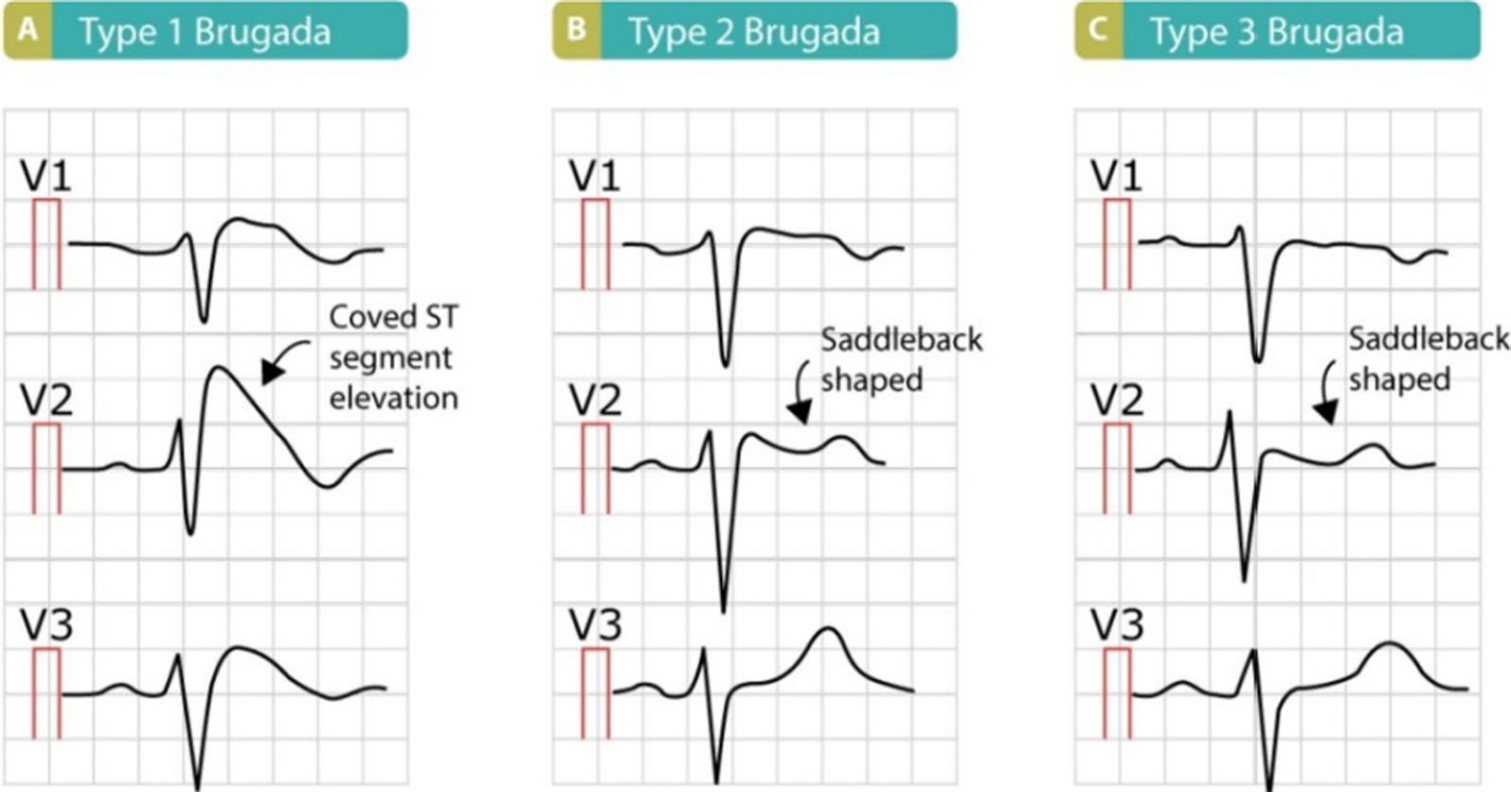
Typical electrocardiogram patterns and classification of Brugada syndrome (https://ecgwaves.com/topic/brugada-syndrome-ecg-treatment-type-1-2-3/)

**Fig. 5:**
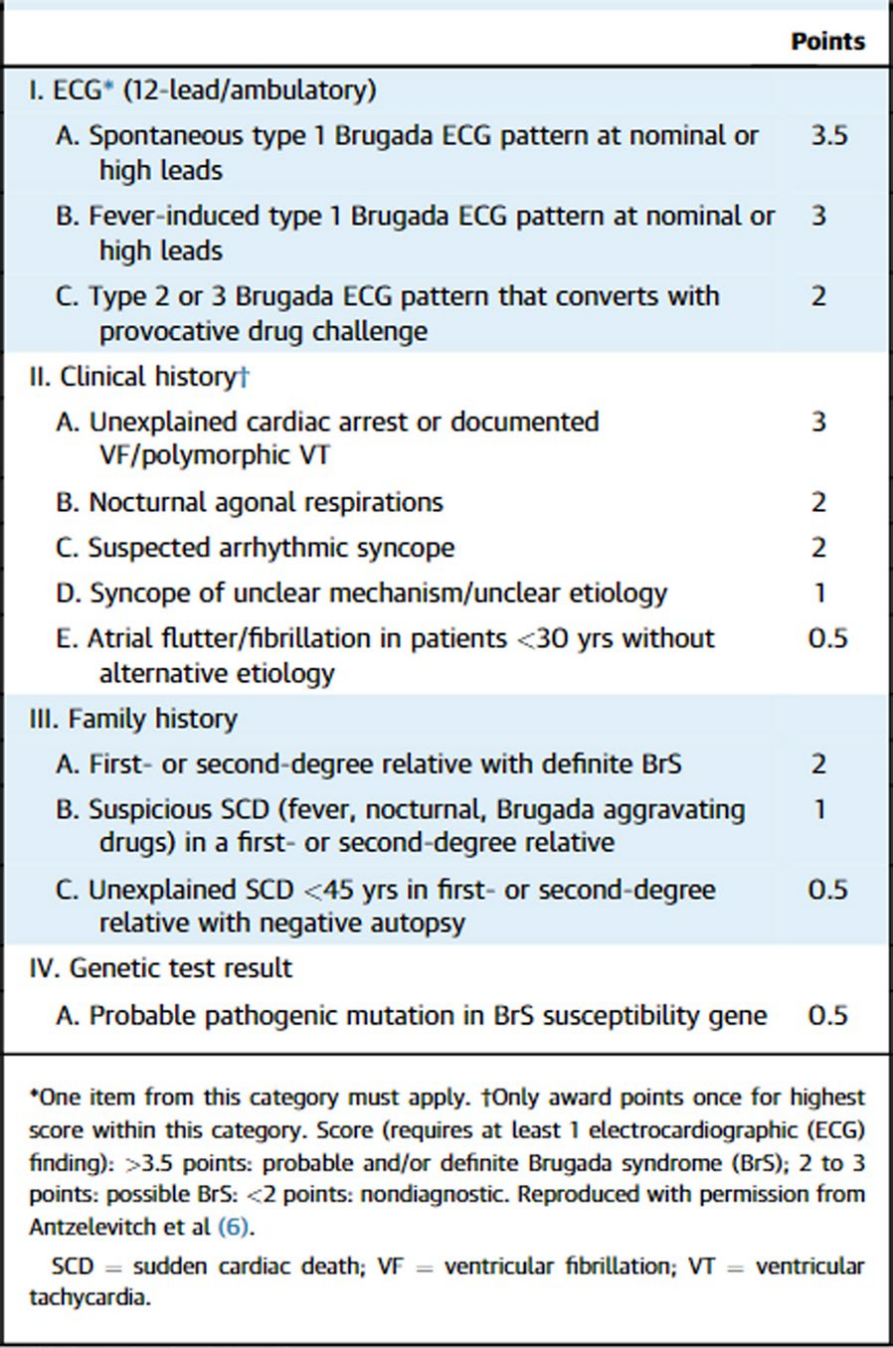
Proposed Shanghai Score System for diagnosis of Brugada syndrome

Diagnosis of Brugada syndrome is typically by electrocardiogram (ECG), however, the abnormalities may not be consistently present [11]. They may be unmasked by administration of sodium channel blockers, for example, ajmaline or flecainide. The characteristic patterns on ECG include coved or saddleback pattern ST elevation and RBBB pattern in V1–V3. These patterns may persist all the time or be elicited in some occasions such as febrile state (as seen in case 1), hyperkalemia, hypokalemia, hypercalcemia, alcohol or cocaine intoxication, and the use of certain medications, including sodium channel blockers, vagotonic agents, α-adrenergic agonists, β-adrenergic blockers, heterocyclic antidepressants, and a combination of glucose and insulin, making the diagnosis much more difficult [12]. According to recent classification, three forms of the Brugada ECG pattern have been described [13] (Fig. 4). Currently, only a type 1 ECG pattern with coved-type ST elevation, either spontaneously or provoked in response to provocation test, can diagnose Brugada syndrome [14]. However, if type I BrS ECG is masked, patients also need to have clinical history, family history, or genetic test result, as in case 1, to meet the latest Shanghai Score System for diagnosis (Fig. 5) [15]. Asymptomatic patients who have these typical ECG features are described as having “Brugada pattern.” The high-risk criteria for patients with BrS who need general anesthesia include [16]: (i) symptomatic cases with syncope or a medical history of VF, (ii) asymptomatic cases showing pathognomonic ST segment elevation on ECG and medication- or EPS-induced VF, and (iii) cases showing coved-type ST elevation on ECGs.

In case 1, the saddleback ECG pattern was non-specific, however, the clinical history raised the possibility of BrS diagnosis. In these kinds of equivocal situations, to increase the sensitivity and specificity of the diagnosis, the ECG lead V1 and V2 can be shifted upward to the second or third intercostal space [17].

### Differential diagnosis

The differential diagnosis for intraoperative ventricular arrhythmias [18, 19] that could lead to hemodynamic compromise includes the conditions in Box 1.

### Box 1. Differential diagnosis of intraoperative ventricular arrhythmias

- Myocardial ischemia or infarction.
- Long QT syndrome (LQTS) with polymorphic VT.
- Catecholaminergic polymorphic VT.
- Brugada syndrome.
- Short QT syndrome.
- Hyperkalemia.
- Hypercalcemia.
- Hypothermia.

In case 1, a young man had no any systemic disease history, preoperation electrolyte imbalance, or hypothermia, and normal ECG, so the non-structural abnormality should be highly suspected [20]. The normal postoperative cardiac enzyme and serial ECG studies rule out the possibility of myocardial ischemia. Brugada syndrome with the characteristic ECG changes was the most probable cause, and later the genetic study confirmed the diagnosis.

### Genetic consideration

Brugada syndrome is an inherited autosomal dominant disease. About a quarter of those patients have an association of family history. More than 100 mutations in seven genes have been associated with BrS. The most commonly involved gene is SCN5A [1]. Loss-of-function mutations in SCN5A, which encodes the α-subunit of the Na(v)1.5 sodium ion channel conducting the depolarizing Na current, causes 15–20% of BrS cases. BrS has a variable gene penetrance; some patients with the same gene mutation may have symptoms, but others may not [18]. In our cases, the variable gene penetrance is obvious; our case 2 patient with confirmed BrS did not have any symptoms during the operation; however, the family history may pose a risk to patients who plan to have surgery [19]. The case 1 patient, on the contrary, did not have any diagnosis of BrS before this event. The diagnosis was not made until positive BrS genetic test appeared during follow-up. Therefore, people with previous history of cardiac arrest without evidence of myocardial infarction need more attention as to possible Brugada syndrome regardless of confirmed diagnosis.

### Perioperative and environmental factors

The abnormal heart rhythms seen in Brugada syndrome often occur at rest, following a heavy meal, or even during sleep. These situations occur when the vagus nerve is activated, and are referred to as periods of high vagal tone [1]. Bradycardia and vagal tone may contribute to ST segment elevation and lethal arrhythmia by decreasing calcium currents [20]. This explains the greater ST segment elevation in high vagal settings and the incidence of ventricular arrhythmias at night. In our cases, the anesthetic state fluctuated with high and low vagal tone, giving it a high tendency of occurrence, and should be carefully managed.

Another common environmental factor affecting the BrS phenotype is temperature. Premature inactivation of the Na(v)1.5 sodium channel has been shown to be accentuated at higher temperatures. This suggests that febrile states as in case 1 may increase the risk of ventricular arrhythmias. Thus, meticulous temperature control is crucial in patients with BrS [21, 22]. Sodium channel blocking medications, such as those used to treat cardiac arrhythmia, may also worsen the tendency toward abnormal heart rhythms. These include class 1A and 1C anti-arrhythmic agents such as amiodarone, verapamil, and adrenergic blockers. There is currently little evidence that these drugs lead to malignant arrhythmias, although they possess the potential to elicit the Brugada ECG pattern [6, 23].

### Treatment and management

There is no specific treatment for Brugada syndrome. Implantable cardioverter defibrillator (ICD) implantation is the only method proved to reduce the incidence of sudden cardiac death [24]. Anti-arrthymic drugs such as amiodarone and β-blocker cannot protect patients from sudden death. In those without symptoms, the risk of death is much lower, and short-term medication may be beneficial [14]. Isoproterenol may be used for those who have frequent life-threatening abnormal heart rhythms, while quinidine may be used longer term as an alternative to ICD [25], although it does not fully suppress the tendency of VF [26, 27].

During anesthesia, the ability of volatile anesthetics to sensitize the myocardium to arrhythmogenic catecholamine has been well known [28], and the volatile anesthetics may be arrhythmogenic due to inhibition of the slow Na^+^ current and induction of reentry of premature, impulses which would potentiate the attack of BrS [29, 30]. Some commonly used anesthetic drugs, such as propofol and bupivacaine, are associated with adverse events in Brugada syndrome [9, 24, 31–35]. Hyper- or hypokalemia and hypercalcemia have all been known to unmask the Brugada ECG pattern [6, 36]. Therefore, perioperative hyperthermia, bradycardia, and electrolyte imbalances should be corrected effectively once occurred in high-risk patients [9]. Intraoperative vagotonic maneuvers (for example, pneumoperitoneum) may cause arrhythmias and should also be avoided [37]. The usage of neostigmine and glycopyrrolate as reversal agents were seen without significant incident. However, it is strongly suggested that it should be given slowly, and the ECG should be monitored during the process due to the possibility of increased vagal tone [13].

## Conclusions

Brugada syndrome, although rare, has the highest incidence in South East Asian young males as in case 1. Careful preoperative evaluation and perioperative management can help reduce the harmful outcome of the disease and prevent any untoward events.

## Data Availability

Not applicable.
